# Ascorbic Acid-A Potential Oxidant Scavenger and Its Role in Plant Development and Abiotic Stress Tolerance

**DOI:** 10.3389/fpls.2017.00613

**Published:** 2017-04-26

**Authors:** Nudrat A. Akram, Fahad Shafiq, Muhammad Ashraf

**Affiliations:** ^1^Department of Botany, Government College University FaisalabadFaisalabad, Pakistan; ^2^Pakistan Science FoundationIslamabad, Pakistan; ^3^Department of Botany and Microbiology, King Saud UniversityRiyadh, Saudi Arabia

**Keywords:** ascorbic acid, biosynthesis, abiotic stress tolerance, AsA and hormone crosstalk, exogenous application

## Abstract

Over-production of reactive oxygen species (ROS) in plants under stress conditions is a common phenomenon. Plants tend to counter this problem through their ability to synthesize ROS neutralizing substances including non-enzymatic and enzymatic antioxidants. In this context, ascorbic acid (AsA) is one of the universal non-enzymatic antioxidants having substantial potential of not only scavenging ROS, but also modulating a number of fundamental functions in plants both under stress and non-stress conditions. In the present review, the role of AsA, its biosynthesis, and cross-talk with different hormones have been discussed comprehensively. Furthermore, the possible involvement of AsA-hormone crosstalk in the regulation of several key physiological and biochemical processes like seed germination, photosynthesis, floral induction, fruit expansion, ROS regulation and senescence has also been described. A simplified and schematic AsA biosynthetic pathway has been drawn, which reflects key intermediates involved therein. This could pave the way for future research to elucidate the modulation of plant AsA biosynthesis and subsequent responses to environmental stresses. Apart from discussing the role of different ascorbate peroxidase isoforms, the comparative role of two key enzymes, ascorbate peroxidase (APX) and ascorbate oxidase (AO) involved in AsA metabolism in plant cell apoplast is also discussed particularly focusing on oxidative stress perception and amplification. Limited progress has been made so far in terms of developing transgenics which could over-produce AsA. The prospects of generation of transgenics overexpressing AsA related genes and exogenous application of AsA have been discussed at length in the review.

## Introduction

Vitamin C [ascorbic acid (AsA)] is an antioxidant molecule and a key substrate for the detoxification of reactive oxygen entities (Smirnoff, 2000; Foyer and Noctor, 2011; Qian et al., 2014). Physiologically active form of AsA is the resonance stabilized anionic form (formed due to deprotonation of the hydroxy group at C_3_) which is termed as ascorbate. Likewise, ascorbate anion (AH^−^) is a vital water-soluble antioxidant molecule within biological system (Buettner and Jurkiewlcz, 1993; Smirnoff, 2000). It is believed that apoplastic ascorbate contents could be vital for environmental stress perception as a direct link and therefore involved in the subsequent downstream stress signaling and responses in plants (Horemans et al., 2000; Zechmann, 2011; Shapiguzov et al., 2012). Cell cytoplasm constitutes the most abundant pool of ascorbate, while to some extent it is also transported across plasma membrane (usually 5%) to the apoplast (Barnes et al., 2002; Zechmann, 2011; Venkatesh and Park, 2012). The apoplastic fraction of AsA is regarded as the determinant of oxidative stress signaling, although its magnitude and duration represent the overall redox status of cell apoplast (Foyer and Noctor, 2005; Gallie, 2013). Generally, the redox buffering capacity of the cell apoplast is low despite the presence of other antioxidant molecules like polyamines and flavonoids (Pignocchi et al., 2003) and nitric oxide (Bethke et al., 2004), and is largely dependent on AsA pools due to the absence of glutathione and NAD(P)H (Foyer and Noctor, 2005). For example, Pignocchi et al. (2006) reported that redox states of the apoplastic ascorbate levels influence hormonal balance, growth responses, MAPK signaling cascades and antioxidant enzyme activities, while glutathione levels remain unaffected. Consequently, due to its apoplastic localization, AsA constitutes a vital role in stress perception, redox homeostasis and subsequent regulation of oxidative stress and plant physio-biochemical responses under normal as well as different abiotic stresses.

Not only this, AsA is a ubiquitious molecule proves effective in improving stress tolerance in plants (Table 1). Most efficient role of exogenously applied AsA is to protect lipids and proteins against salinity or drought-induced oxidative adversaries (Miguel et al., 2006; Khan et al., 2010; Naz et al., 2016). It can improve tolerance against abiotic stresses by enhancing plant growth, rate of photosynthesis, transpiration, oxidative defense potential and photosynthetic pigments (Table 1). Khan et al. (2006) observed that exogenous application of AsA (50 and 100 mg L^−1^) improved chlorophyll “*a*” contents of wheat seedlings subected to salt stress. Similarly, an increase in plant growth and proline content and decrease in ion leakage and lipid peroxidation was observed in okra plants under drought stress (Amin et al., 2009). Athar et al. (2009) observed the influence of AsA (100 mg L^−1^) via seed soaking, rooting medium and foliar spray on wheat plants under salinized hydroponic culture. They found that AsA application improves growth, AsA content, and activities of superoxide dismutase (SOD), peroxidase (POD) and catalase (CAT) antioxidative enzymes.

**Table 1:** **Improvement in growth and different physio-biochemical attributes by exogenous application of ascorbic acid (AsA) in different species under stress conditions**.

**Mode of AsA application**	**AsA level**	**Plant species**	**Effects**	**References**
Pre-sowing treatment	50 mg L^−1^	*Lens* culinaris Medik.	AsA improved yield and yield components under salinity stress	Alami-Milani and Aghaei-Gharachorlou, 2015
	100 and 200 mg L^−1^	Sunflower (*Helianthus annuus* L.)	AsA significantly enhanced germination rate, germination percentage, plumule length and seedling fresh biomass under drought stress	Ahmed et al., 2014
	1 and 2 mM	Sunflower (*H. annuus* L.)	AsA enhanced germination rate, germination percentage, seed stamina index and fresh and dry weights under drought stress	Fatemi, 2014
	1 mM	Barley (*Hordeum vulgare* L.)	AsA improved proline, RWC, chlorophyll, enzymatic antioxidants and leaf anatomy under NaCl stress	Agami, 2014
	1.5, 1.0, 2.0 and 4.0 mM	Faba bean (*Vicia faba* L.)	AsA decreased micronucleus frequency and chromosomal aberration, while it improved mitotic index under Pb stress	Yu et al., 2014
	0.25, 0.5 and 1.0 mM	Sugar cane (*Saccharum officinarum* L.)	AsA significantly enhanced fresh weight, number of shoots/roots, shoot/root length, soluble protein contents and enzymatic antioxidants	Munir et al., 2013
	55, 110 and 165 μM	Safflower (*Carthamus tinctorius* L.)	AsA improved germination percentage, seedling fresh and dry weights, shoot and root lengths and vigor index under salt stress	Razaji et al., 2012
	15 and 30 mg L^−1^	Squash (*Cucurbita maxima* D.)	AsA improved seedling growth, fresh and dry matter, protease activity and chlorophyll contents under salinity stress	Rafique et al., 2011
Foliar spray	500, 1000 and 2000 mg L^−1^	Olive (*Olea europea* L.)	Plant height, leaf number, leaf area and lateral shoot number were enhanced by AsA	Mayi et al., 2014
	200 mg L^−1^	Wheat (*Triticum aestivum* L.)	AsA enhanced chlorophyll *a* and *b*, total soluble proteins, carbohydrates and carotenoids under drought stress	Hussein et al., 2014
	150 mg L^−1^	Pearl millet (*Pennisetum glaucum* L.)	AsA significantly enhanced leaf area and number of leaves	Hussein and Alva, 2014
	100, 200 and 300 mg L^−1^	Chickpea (*Cicer arietinum* L.)	Plant height, seed yield and harvest index were improved by AsA	Zarghamnejad et al., 2014
	50, 100 and 150 mg L^−1^	Canola (*Brassica napus* L.)	AsA improved shoot and root fresh weights, root dry weight, qN, NPQ, shoot and root P and AsA contents under drought stress	Shafiq et al., 2014
	500 mg L^−1^	Wheat (*T. aestivum* L.)	AsA increased growth, grain yield and yield components	Mohamed, 2013
	100 and 200 mg L^−1^	Sunflower (*Helianthus annuus* L.)	AsA improved stearic acid, linoleic acid and palmitic acid percentage and oil yield under drought conditions	Ahmed et al., 2013
	100, 200 and 300 mg L^−1^	Wheat (*T. aestivum* L.)	AsA increased number of tillers and spikes per plant, spike length, spikelets/spike, and grain and straw yield under reclaimed sandy soil	Bakry et al., 2013
	75 and 150 mg L^−1^	Maize (*Zea mays* L.)	AsA significantly enhanced RWC, seed yield and chlorophyll contents under water deficit conditions	Darvishan et al., 2013
	0.1, 0.5 and 1 mM	*Saccharum* spp.	POD, SOD proline contents and growth improved by AsA application while protein decreased under salt stress	Ejaz et al., 2012
	50, 100 and 150 mM	Sunflower (*H. annuus* L.)	AsA decreased flavonoids, anthocyanins and total soluble sugars under water deficit conditions	Ebrahimian and Bybordi, 2012
	400 and 600 mg L^−1^	*Camellia* spp.	AsA improved chlorophyll *a* and *b*, polyphenol oxidase activity, phenylalanine ammonia lyase activity and brewed tea liquor characteristics	Murugan et al., 2012
	0.7 mM	Wheat (*Triticum durum* L.)	AsA improved leaf area, chlorophyll and carotenoid contents and proline, while it decreased H_2_O_2_under salt stress	Azzedine et al., 2011
	1 and 3 mM	Savory (*Satureja hortensis*)	AsA enhanced growth, proline and soluble proteins under drought stress	Yazdanpanah et al., 2011
	0.7 mM	Wheat (*Triticum durum* L.)	AsA significantly improved chlorophyll and carotenoid contents, leaf area, and proline, and it decreased H_2_O_2_ under salinity stress	Fercha et al., 2011
	100 mg L^−1^	Wheat (*T. aestivum* L.)	AsA enhanced antioxidant enzyme activities, ascorbate, phenol, carotenoids, potassium, calcium, magnesium as well as mitigated the adverse effects of salinity on leaf senescence	Farouk, 2011
	100, 150 and 200 mg L^−1^	Basil (*Ocimum basilicum* L.)	Fresh and dry weights, RWC, photosynthetic pigments, growth and oil percentage were improved under water stress	Khalil et al., 2010
	200 and 400 mg L^−1^	Faba bean (*Vicia faba* L.)	AsA increased total carbohydrates, proteins and solute concentration as well as enhanced Mg^2+^, Ca^2+^, P and K under salinity stress	Sadak et al., 2010
	50, 100 and 150 mg L^−1^	Maize (*Zea mays* L.)	AsA significantly increased stem and leaf dry weights and leaf fresh weight as well as grain weight under water deficit conditions	Dolatabadian et al., 2010
	50 and 100 mg L^−1^	Shoe flower (*Hibiscus rosasinesis* L.)	AsA improved fresh and dry weights, number of flowers/plant, carotenoids, chlorophyll *a* & *b*, soluble sugars, nitrogen, phosphorus and potassium contents	Fatma et al., 2009
	100 mM	Common bean (*Phaseolus vulgaris* L.)	AsA improved chlorophyll contents and decreased ABA under salinity	Dolatabadian et al., 2009
	1 mM	Okra (*Hibiscus esculentus* L.)	AsA significantly increased fresh and dry weights, sugar contents, proline, chlorophyll *a* & *b*, carotenoids and leaf area under drought stress	Amin et al., 2009
	25 mM	Canola (*Brassica napus* L.)	AsA decreased activities of antioxidant enzymes and MDA in leaf, and improved protein contents under salinity stress	Dolatabadian et al., 2008
	200 and 400 mg L^−1^	*Khaya senegalensis*	AsA improved chlorophyll *a, b* and carotenoid contents, total sugars and uptake of P, K and N contents under salinity stress	Nahed et al., 2006
	50 and 100 mg L^−1^	Wheat (*T. aestivum* L.)	AsA improved chlorophyll *a* contents and Na^+^ accumulation under drought stress	Khan et al., 2006
Foliar and pre-sowing	20 and 40 mg L^−1^	Maize (*Zea mays* L.)	AsA enhanced seedling growth, chlorophyll *b*, leaf relative water content, membrane stability and activities of enzymatic antioxidants at low temperature	Ahmad et al., 2014
	1 mM	Wheat (*Triticum aestivum* L.)	AsA maintained net photosynthesis, chlorophyll contents and growth under drought stress	Malik and Ashraf, 2012
	100 mg L^−1^	Milk thistle (*Silybum marianum* L.)	AsA enhanced seed germination. growth, carotenoids, plant water status, AsA, antioxidant enzyme activities and protein bands under salinity stress	Ekmekçi and Karaman, 2012
	50 and 100 mg L^−1^	Sorghum (*Sorghum bicolor* L.)	AsA improved germination percentage, thickness of xylem and phloem tissues and leaf blade under saline conditions	Arafa et al., 2009
	100 mg L^−1^	Wheat (*Triticum aestivum* L.)	AsA enhanced growth, CAT, POD and SOD activities and photosynthetic rate under saline conditions	Athar et al., 2009
	50 and 150 mg L^−1^	Wheat (*Triticum aestivum* L.)	Foliar and presowing treatment enhanced CAT, K, Ca^2+^, photosynthetic pigments, AsA contents, while foliar spray also improved growth	Athar et al., 2008

Genetic engineering to improve abiotic stress tolerance is one of the important strategies being under consideration these days. A variety of genetically engineered crop plants have been generated particularly with superior salinity and drought tolerance. Potentially, AsA can improve plant growth and yield as a potential regulator of different mechanisms under adverse factors, so over-accumulation of AsA in plants through gene (s) engineering could efficiently increase plant stress tolerance. Yet, not a single report in the literature is available on stress tolerant genetically modified plants with high accumulation of AsA. Thus, under stress conditions the genetic manipulation of plants with the goal of obtaining high accumulation of AsA is an essential area to be considered. Thus, the present review focuses on how biosynthesis of AsA is regulated in plants under stress or non-stress conditions and how far AsA accumulation in plants has been improved by different means.

## Biosynthesis of ascorbic acid

It took almost more than a decade to completely elucidate the intriguing pathways of ascorbate biosynthesis in plants. However, such efforts have led to identify several important enzymes and key intermediates. The biosynthesis of AsA in higher plants takes place in mitochondria via several proposed routes. The **primary most elucidated pathway** is the Smirnoff-Wheeler pathway which is also called as D-mannose/L-galactose pathway (Wheeler et al., 1998). Briefly during this pathway, D-glucose is converted to D-glucose 6-phosphate by the enzyme hexokinase. The D-glucose 6-phosphate formed is then converted to GDP-D-mannose via four steps reversible process catalyzed by the enzymes phosphogluco-isomerase, mannose 6-phosphate isomerase, phosphomannose mutase and GDP-D-mannose phosphorylase, respectively. The second major phase involves the conversion of GDP-D-mannose formed previously into GDP-L-galactose during three reactions catalyzed successively by GDP-D-mannose 3′,5′-epimerase, GDP-L-galactose phosphorylase and L-galactose 1-P phosphatase. The GDP-L-galactose is subsequently converted to L-galactose by the action of enzyme L-galactose dehydrogenase and finally into L-galactono-1, 4-lactone (final precursor of AsA). Lastly, ascorbic acid is formed from L-galactono-1,4-lactone in an enzymatic reaction catalyzed by L-galactono-1,4-lactone dehydrogenase. The second **important pathway** involves the cell wall pectins. The degradation results in the formation of methyl-galacturonate which is converted into L-galactonate via two reactions catalyzed by methyl esterase and D-galacturonate reductase. Later on, the enzyme aldono lactonase catalyze the conversion of L-galactonate into L-galactono-1, 4-lactone and is finally used in ascorbate synthesis (Smirnoff et al., 2001).

**Another reported pathway** involves the conversion of GDP-D-mannose to GDP-L-gulose and subsequent formation of L-gulono-1, 4-lactone via L-gulose (Wolucka and van Montagu, 2003). This is similar to the primary pathway which starts from glucose however this pathway branch off from GDP-D-mannose. From here, GDP-D-mannose is converted to L-gulose in three reactions catalyzed by GDP-D-mannose3′, 5′-epimerase, GDP-L-gulose-1-P-phosphatase and L-gulose-1-P-phosphatase, respectively. At this point, the enzyme L-gulono-1, 4-lactone dehydrogenase catalyze the conversion of L-gulose into L-gulono-1, 4-lactone which is then finally converted to AsA (Smirnoff et al., 2001).

**Figure 1:**
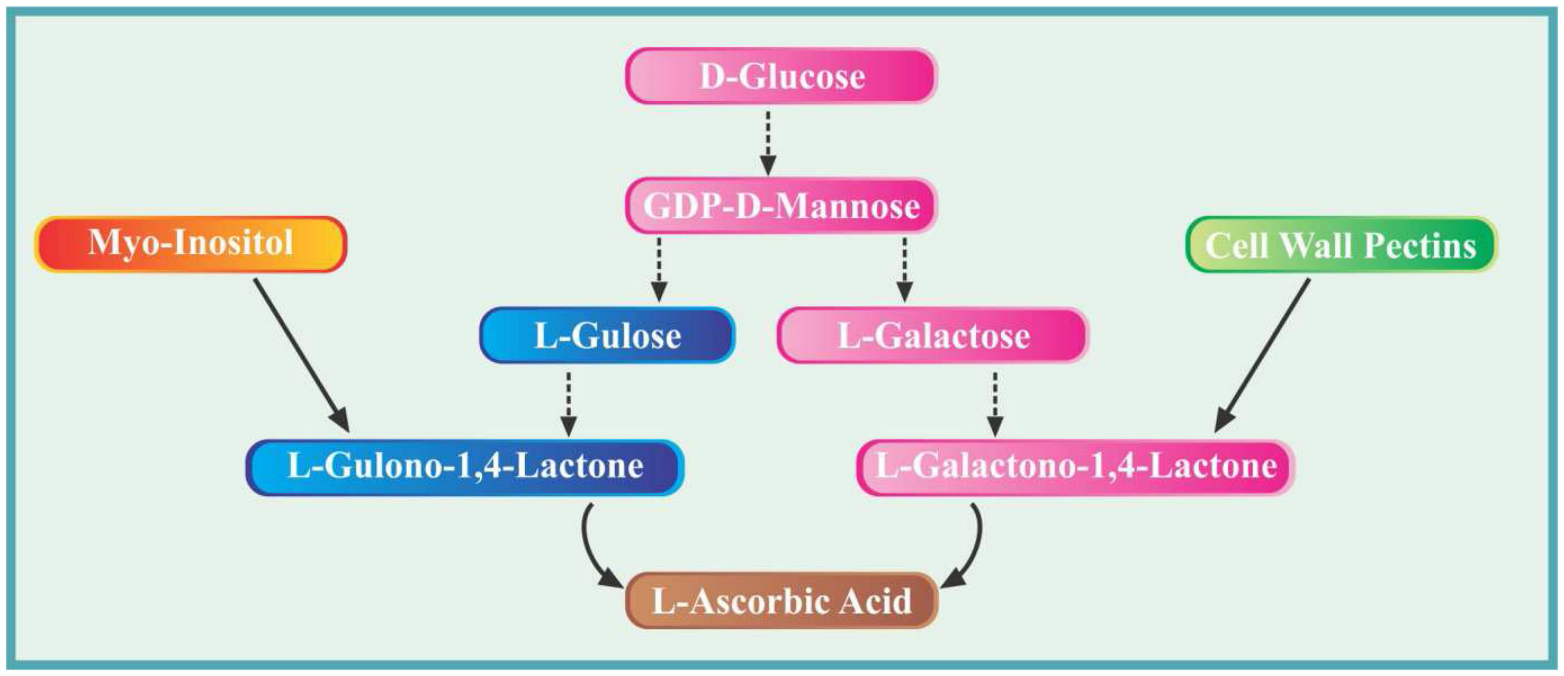
**Diagram showing various pathways and important precursor molecules involved in biosynthesis of ascorbic acid in plants**.

In a **fourth reported pathway**, the synthesis of ascorbate from myo-inositol is reported. Briefly, myo-inositol is converted to L-gulono-1, 4-lactone via three reactions catalyzed by myo-inositol oxygenase, glucuronate reductase and aldono lactonase (Valpuesta and Botella, 2004). The gulono-1, 4-lactone is finally used in ascorbate synthesis (Smirnoff et al., 2001). For convenience, major pathways and important precursor molecules have been elucidated in the form of a schematic diagram (Figure 1).

## Ascorbate role in oxidative defense metabolism

Due to the regenerative nature of ascorbate, it is one of the most powerful antioxidant molecules (Noctor and Foyer, 1998) which along with vitamin E plays a key role in quenching intermediate/excited reactive forms of molecular oxygen either directly or through enzymatic catalysis (Noctor and Foyer, 1998; Ozdemir et al., 2004; Ye et al., 2012). In addition, the ascorbate-glutathione pair is involved in regulation of plant development processes through the manipulation of oxidative metabolism (Asada, 1999; Pignocchi and Foyer, 2003). Apart from this, ascorbate maintains ROS levels within tolerable capacity (Kocsy et al., 2001; Shao et al., 2008).

The antioxidant significance of ascorbate-glutathione cycle in redox active organelles like mitochondria and peroxisomes is well reported. The ascorbate peroxidase-glutathione reductase (APX-GR) system is far efficient in detoxification of hydrogen peroxide (H_2_O_2_) than catalase (CAT) and peroxidase (POD) and the exogenous addition of ascorbate through the nutritional solution was reported to result in marked reduction in the activities of both enzymes (Dolatabadian and Jouneghani, 2009). In this connection, the involvement of Halliwell-Asada (ascorbate-glutathione) pathway in neutralizing H_2_O_2_ is widely discussed (Bray et al., 2000). Pavet et al. (2005) investigated the relationship of glutathione and AsA concentrations in the activation of plant innate stress responses through antioxidative metabolism and it was concluded that elevated levels of glutathione, its reduced form, and ascorbate deficiency triggers salicylic acid (SA) mediated signaling that is independent of H_2_O_2_. Indirect evidence also supports the antioxidant potential of AsA molecule. The elevated doses of exogenous ascorbate can inhibit germination through ROS elimination which results in failure of germination initiation (Takemura et al., 2010). The distinct nature of ascorbate allows it to be part of enzymatic and non-enzymatic antioxidant defense system and thereby increased efficiency and contribution to ROS neutralization and balance. This makes AsA as a fragile substrate in plant developmental and stress responses.

It is believed that ascorbate can effectively regulate antioxidative metabolism in plants (Anjum et al., 2014; Noctor et al., 2014) and endogenous AsA levels can be improved by exogenous supply of AsA via foliar spray, seed pre-treatment and its application through rooting medium (Athar et al., 2008). Several studies report the AsA-regulation of antioxidant defense metabolism in different plants grown under stress conditions, e.g., canola under salinity (Bybordi, 2012), *Abelmochus esculentus* under salinity (Raza et al., 2013), *Hordeum vulgare* under salinity (Agami, 2014), *Brassica napus* under drought (Shafiq et al., 2014), etc. This has been mainly ascribed to AsA involvement in activation of different antioxidant enzymes. Of different antioxidative enzymes known to be involved in antioxidant defense system, ascorbate peroxidases (APXs) are heme containing enzymes that are known to dismutase H_2_O_2_ to water and molecular oxygen using AsA as an electron source (Shigeoka et al., 2002; Mittler et al., 2004; van Doorn and Ketsa, 2014). It is further reported that APX gene expression is affected by abiotic stresses like salinity, drought, light fluctuations and abcisic acid (ABA) production (Bonifacio et al., 2011; Caverzan et al., 2012; Nam et al., 2012; Ben Rejeb et al., 2014) and this effect is differentially modulated among plants depending upon the nature, duration and intensity of a stress and plant type (Caverzan et al., 2012). Nonetheless, APXs are often characterized primarily due to their subcellular localization (Shigeoka et al., 2002; Caverzan et al., 2012; Noctor et al., 2014; Ben Rejeb et al., 2014). Previously, Nakano and Asada (1981) attributed ROS detoxification in chloroplast to the ascorbate-glutathione system. Even so, APX and ascorbate-glutathione cycle is also involved in ROS stabilization in peroxisomes, mitochondria and cell cytosol (Mittler et al., 2004; Anjum et al., 2014; Noctor et al., 2014). Various isoforms of APX have been characterized based upon their specific function. For instance, the enzymatic function of APX1 and APX2 is well studied among the three cytosolic APXs (Suzuki et al., 2013). APX1 which expresses in Arabidopsis roots is a cytosolic enzyme that catalyzes H_2_O_2_ detoxification using AsA as an electron source (Fourcroy et al., 2004; Noctor et al., 2014). The role of APX1 in regulating oxidative stress is very much focused in the recent studies (Maruta et al., 2012; Ben Rejeb et al., 2014; Zhang et al., 2015). It is the most abundant APX isoform that shows a protective role and regulates ROS signals (Suzuki et al., 2013). The co-expression of APX1 and APX2 under abiotic stresses resulted in regulation of H_2_O_2_ contents and subsequent oxidative stress (Noctor et al., 2014).

In addition to the role of APX1 in the light harvesting compartments, the role of APX2 in the bundle sheath cells has also been proposed (Suzuki et al., 2013; Gorecka et al., 2014). In chlorophyll fluorescence based study, Fryer et al. (2003) reported the expression of APX2 in response to photosynthetic electron chain mediated localized production of H_2_O_2_. Furthermore, the antioxidant role of APX2 under abiotic stress conditions like salinity, heat and light stress has also been reported (Ren et al., 2010; Jung et al., 2013; Suzuki et al., 2013). More recently, using Arabidopsis knockout lines, Chen et al. (2014) have reported the protective role of APX6 (a new form of APX) in maintaining seed vigor under stress-induced excessive oxidative stress. For the first time, interplay between ABA and ROS was discovered in the form of a new ROS signal, so its interdependence with auxin and ROS interaction has been proposed (Chen et al., 2014). The diverse involvement of all three APX isoforms in ROS signal regulation indicates the key role of ascorbate in antioxidant metabolism in most plants. It is likely that with the discovery of some new isoforms of APXs in future, the researchers will be able to reinforce the interplay between APXs and ascorbate in plants exposed to stressful environments.

## Apoplast ascorbate levels and expression of ascorbate oxidase

A substantial portion of cellular AsA (almost 90%) is reported to be localized in the cytoplasm, however, in apoplast its concentration is reported in milli-molar range due to its export from the cytosol (Pignocchi et al., 2003; Smirnoff, 2005). This extracellular ascorbate constitutes a primary defense line against external oxidants (Plöchl et al., 2000; Zechmann, 2011; Anjum et al., 2014; Kangasjarvi and Kangasjarvi, 2014).

Ascorbate oxidase (AO) is a glycoprotein and a member of blue copper oxidase family that mediates ascorbate oxidation to dehydroascorbate (DHA) which usually follows high affinity transport across the membrane (Smirnoff, 2000; Szarka et al., 2004; Barbehenn et al., 2008; De Tullio et al., 2013). Dehydroascorbate (oxidized form) in the apoplast is then transported into cell cytosol and is exchanged with reduced form to ensure constant apoplast redox flux (Horemans et al., 2000; De Tullio et al., 2013; Anjum et al., 2014). In cytoplasm, DHA reductase (DHAR) using glutathione as an electron donor promotes the reduction of DHA to AsA (Ioannidi et al., 2009; Gallie, 2013). The physiological significance of the two oxidized forms of AsA viz. monodehydroascorbate and dehydroascorbate has been extensively reported in the past (Noctor and Foyer, 1998; Stevens et al., 2008; De Tullio, 2012; Gallie, 2013; Anjum et al., 2014; Pandey et al., 2015).

Interestingly, the proper function of this enzyme remained unexplained for many years. By being localized in cell wall, AO is a key determinant of apoplast redox status (Kato and Esaka, 2000; Fotopoulos and Kanellis, 2013), hence it is involved in growth, development, stress perception and subsequent stress signaling (Pastori and Foyer, 2002; Foyer and Noctor, 2005, 2013; Sanmartin et al., 2007; De Tullio et al., 2013). Furthermore, light and hormonal mediated regulation of AO expression with its role in plant growth modulation has also been proposed (Pignocchi et al., 2003; Gallie, 2013). For example, a 40-fold increase in the AO activity resulted in improved elongation and biomass production in *Nicotiana tobaccum* while reduction was evident when the authors implemented AO antisense approach (Pignocchi et al., 2003). Oxidative stress can also induce the expression of AO (Sanmartin et al., 2003) and this shows its peculiar involvement in oxidative stress regulation (De Tullio, 2010). However, contrasting reports regarding its effect on biomass production are also available (Sanmartin et al., 2003; Yamamoto et al., 2005). In another study, increased salinity stress tolerance was evident upon inhibition of AO expression transgenic tobacco and arabidopsis plants (Yamamoto et al., 2005). In a recent study, Fotopoulos and Kanellis (2013) reported reduced apoplast redox status in transgenic tobacco plants over-expressing AO enzyme, however, it was linked with delayed dark induced senescence through acquired tolerance due to higher H_2_O_2_ levels in the apoplast. Since AO activity results in the oxidation of ascorbate and depletes apoplastic ascorbate pool and decreases redox status of the cell wall. And the enhanced AO activity has been linked with decreased stress tolerance (Garchery et al., 2013).

Interestingly, the function of this enzyme in the past was often confused or neglected in several studies, so it requires to be elucidated well. The extracellular localization of this enzyme proposes its role as a link between environmental stress perception and adaptive response. Recently, in this connection, De Tullio et al. (2013) proposed the role of AO in the stress perception and downstream signaling events. At present, AO role in several events can be integrated: (1) regulation of apoplast redox status, (2), increased H_2_O_2_ accumulation in the apoplast similar to action of NADPH oxidase, (3) the involvement of DHA in signaling events, and (4) possible involvement of (APX) in the apoplast.

## Comparative role of AO and APX in plant cell apoplast

Striking functional similarities exists for (APX) and (AO) and the role of both these ascorbate related enzymes within plant growth and development is imperative. Both AO and APXs are responsible for the conversion of reduced form of ascorbate to an oxidized form (dehydro-ascorbate) which is then transported to cytoplasm for subsequent glutathione mediated reduction to ascorbate finally recycled back to cell apoplast. The biochemical aspects of these two enzymes have been considered in the previous paragraphs in detail while some aspects are considered again. How both of these two enzymes determine substrate specificity or how their regulation takes place can provide insight for discrimination/identification of developmental and stress responses. Here we propose cross talk between AO and APX which can be further integrated with hormonal network.

As discussed previously, AO through oxygen initiates ascorbate oxidation in apoplast and this phenomenon is reported to be linked with cell expansion, cell division (Joo et al., 2001; Sanmartin et al., 2007) and fruit expansion (Ioannidi et al., 2009). Pignocchi et al. (2003) proposed light induced up-regulation of AO expression that can contribute to cell wall expansion through its interaction with growth hormone auxin. Likewise, auxin and AO interactions have also been reported recently and urge exploration (Kerk et al., 2000; Pignocchi et al., 2006; Bartoli et al., 2013). Consequently, AO effects on growth might involve three key regulating systems viz. light, redox status of the apoplast along with growth hormone, auxin (De Tullio et al., 2004, 2007). The involvement of AO in various developmental processes has been comprehensively reported (see review article by De Tullio et al., 2013).

On the other hand, APX also results in the oxidation of ascorbate to monodehydro-ascorbate (MDHA) which finally decomposes rapidly to dehydro-ascorbate (DHA) (Smirnoff, 2000; Venkatesh and Park, 2014; van Doorn and Ketsa, 2014). However, in contrast to AO, here the reduction of hydrogen peroxide takes place and again ascorbate is the electron donor in this reaction (Teixeira et al., 2004). At this point, both AO and APX are responsible for the oxidation and subsequent apoplast ascorbate pool and its redox status but the electron acceptors are different i.e., O_2_ in the former and H_2_O_2_ in the later reaction. The degradation mechanism for AsA has already been proposed (Parsons and Fry, 2012).

In this context, increased salt tolerance was reported in arabidopsis and tobacco plants with suppressed expression of the apoplastic AO gene (Yamamoto et al., 2005). Similar reports of increased abiotic stress tolerance consistent with decreased AO activity in tomato (Garchery et al., 2013). The higher ascorbate pool in the absence of AO activity was attributed to oxidative stress tolerance (Yamamoto et al., 2005). It is further proposed that AO affect AsA/DHA which might lead to the induction of APX enzyme (Yamamoto et al., 2005). Alteration in AsA/DHA ratio is involved in stress sensing (Pignocchi and Foyer, 2003; Pignocchi et al., 2003; Fotopoulos et al., 2008; De Tullio et al., 2013) and how subsequent signaling cascades lead to the induction of APX is an intresting and intringuing aspect. Not only this, AO is an important determinant of apoplast redox status and the leaf apoplast redox status specifically modulate plant growth and response to hormones, antioxidant enzyme activities, epression patterns of catalase, glycolate oxidase and some other genes, MAPK activity and regulation of transcripts associated with calcium channels (Pignocchi et al., 2006). Based on these available reports, we propose that there could be some kind of regulatory mechanisms between apoplast AO and APX through feedback mechanism which might also involves cross talk with hormones, ROS and NADPH oxidases.

Plant NADPH oxidases are recently characterized for the amplification of ROS signal in form of H_2_O_2_ which spreads in both directions (Takeda et al., 2008; Mittler et al., 2011). There is a need to draw parallels between the comparative role of AO and APX under normal and stress conditions. Although the role of AO and APX follow different mechanisms, but the substrate for both the apoplast localized enzymes is common. Both enzymes are responsible for the oxidation of extracellular apoplastic pool and subsequent regulation of apoplast redox status. The fact that H_2_O_2_ production is enhanced under stress conditions, it will be interesting to evaluate how the activities of AO and APX enzyme are comparatively regulated and to what extent they contribute to stress perception and downstream signaling events.

## Ascorbate-hormone crosstalk

Apart from the role in physiological functioning of plants (Wasternack and Hause, 2002; Spaepen, 2015), regulation of developmental plasticity and adaptive features in plants under normal and stressful environmental conditions is hormone dependent (Ashraf et al., 2010). The survival of plants under stress is reliant on their ability to perceive and convey the environmental stimulus in the form of signals and finally adjust themselves accordingly through biochemical or developmental modifications to ensure continued existence (Hasegawa et al., 2000; Dhawan and Sharma, 2014). The signaling cascades and pathways are generally very integrative in nature and are primarily regulated by hormones (Atkinson and Urwin, 2012; Lima-Silva et al., 2012). For this, the involvement of MAPK cascade (mitogen activated protein kinases) for transmitting hormone and ROS signaling events is elucidated (Fujita et al., 2006) and ideas about hormone crosstalk are supported at molecular level (Bajguz and Hayat, 2009; Rodriguez-Villalon and Hardtke, 2014; Anwar et al., 2015).

Generally, under stress conditions, plant hormonal signaling results induction of various acclimation responses, ultimately regulating plant physio-biochemical processes (Boursiac, 2008; Mittova et al., 2015). Hormonal and ROS signaling networks are highly integrated and both collectively induce appropriate responses under stress by processing and transmitting environmental inputs (Baxter et al., 2014). As the signaling pathways in response to a particular stress can antagonize or interact with each other and the events are principally regulated by hormones (Anderson et al., 2004; Atkinson and Urwin, 2012; Kuromori et al., 2014), the involvement of phyto-hormones in signal transduction leading to overall stress response is therefore possible (Pospisilova et al., 2005; Tuna et al., 2008; Pieterse et al., 2009; Colebrook et al., 2014). Moreover, hormones can also regulate key osmo-protectants thereby contributing significantly to stress tolerance (Pospisilova, 2003; Golldack et al., 2014). Several studies report hormone-induced regulation of defense responses and signaling in plants (Van Loon et al., 2006; Iqbal and Ashraf, 2007; Pieterse et al., 2009; Siddiqui et al., 2011; Golldack et al., 2014; Spaepen, 2015). In this review, some of these complex interactions among different hormones involving ascorbate metabolism are considered in the follow-up passages so as to draw an appropriate sketch of ascorbate-hormone integrated control of plant development.

The apoplast ascorbate pool and its redox state modulate gene expression subsequently resulting in altered hormone-mediated plant responses (Pignocchi et al., 2006; Dinler et al., 2014). It is further reported that endogenous levels of ascorbate affect biosynthesis of different hormones and subsequent hormone-mediated signal transduction under stress conditions (Pastori et al., 2003). For example, ascorbate level in tomato plants was positively correlated with activation of genes involved in hormone signaling as compared to ascorbate biosynthesis which in turn was dependent on oxidative status (Lima-Silva et al., 2012). Sadak et al. (2013) studied that application of AsA and citric acid (2:1) resulted in increased levels of gibberellic acid (GA), indole acetic aid (IAA), zeatin and brasinosteroids (BR), and decreased abscisic acid (ABA) contents. Analogous to this, Ye et al. (2012) reported a possible inter-play between ascorbate and phyto-hormones in *Oryza sativa*. Since AsA is a co-factor required for the biosynthesis of gibberellins, ethylene and abscisic acid (Barth et al., 2006; Ye et al., 2012; Zhang, 2013a,b), cellular ascorbate levels through their interaction with phytohormones influence various signal transduction pathways to regulate growth and development (Pastori et al., 2003). Thus, it is likely that the interplay between AsA and phyto-hormones determines oxidative stress regulation and subsequent developmental responses in plants.

In this context, regulation of ABA synthesis and its subsequent accumulation was proposed to be regulated by redox signal coupled by AsA and DHA levels (Chen et al., 2014). Similarly, brassinosteroids mediated accumulation of H_2_O_2_ in the apoplast led to an increase in the activities of antioxidant enzymes in *Cucumis sativus* (Jiang et al., 2012). Consequently, brassinosteroid-induced stress tolerance was linked with NADPH oxidases which result in H_2_O_2_ build up in the cell apoplast and which subsequently further regulates ABA levels to induce prolonged stress response (Zhou et al., 2014). Interestingly, BR biosynthetic mutants with lower BR levels were associated with AsA/DHA and GSH/GSSG ratios and these were improved upon exogenous BR application which in turn also improved these ratios (Zhou et al., 2014). These reports clearly suggest the crucial involvement of AsA in regulating plant stress responses. For proper understanding, some other process involving ascorbate-phytohormone cross talk is likely to be uncovered in future.

The interplay between ascorbate and different plant hormones is very prominent in **seed germination**. For example, Ye et al. (2012) showed that both ROS and ascorbate play an imperative role in modulating phytohormone levels during seed germination. Elevated ABA levels inhibit ascorbate biosynthesis which in turn suppresses GA levels (Ye et al., 2012) while in contrast, ROS levels are reported to induce biosynthesis of GA as in Arabidopsis through enhanced gene expression (Liu et al., 2010). In addition, enhanced ABA levels in seeds reduce ROS levels in the aleurone layers which suppress AsA biosynthesis, subsequently restraining GA production and amylase activity (Ye et al., 2012). Alternatively, reduction in ABA concentration will lead to enhanced ROS and ascorbate production, there by resulting in enhanced GA levels. Higher GA levels in seeds can then induce the gene expression required for the initiation of seed germination and seedling establishment. Basically, ABA inhibits the activity of the plant NADPH-oxidases which are the key enzymes for ROS production during seed germination (Ye et al., 2012).

Like in seed germination, a controlled link between ascorbate and hormones is evident in fruit **expansion and ripening**. Ascorbate in the apoplast generates ROS which promote fruit ripening (Green and Fry, 2005; Gapper et al., 2013). The ROS so generated result in polysaccharide degradation which causes fruit softening (Dumville and Fry, 2003). Parallel to this, higher AO activity during fruit development contributes to fruit expansion while this declines during ripening, thereby resulting in more reduced AsA contents (Ioannidi et al., 2009). At this stage, the key intermediates involved in the ascorbate biosynthesis contribute to induction of ethylene (Ioannidi et al., 2009) which is further involved in the regulation of steps involved in subsequent fruit ripening (Gapper et al., 2013).

Furthermore, **senescence** is directly linked with increased ROS levels due to inadequate antioxidant capacity in most plants (Zimmermann and Zentgraf, 2005). Like other antioxidants, lower ascorbate pools in the cell contribute to increase ROS that can considerably damage the photosynthetic apparatus, leading to a faster decline in photosynthetic activity in AsA-deficient tissues, thereby speed up senescence (Barth et al., 2006).

It is well aasumed that ascorbate-hormone interaction plays a pivotal role in regulation of senescence (Zhang, 2013b). This assumption has been confirmed by various studies using ascorbate deficient vtc1 mutants and it was linked with the modulation of ABA (Zhang, 2013b). Higher ABA contents reported in vtc1 mutants than wild type were also reported earlier (Pastori et al., 2003). Basically AsA influence the expression of senescene associated genes (SAGs) and lower AsA fraction is linked with increased senescence while higher vitamic C contents delayed it (Zhang, 2013b). The findings have been confirmed in detached leaves of vtc1 mutants which lost chlorophyll contents rapidly than wild type plants (Zhang, 2013b). In agreement, the early expression of *SAG13* and *SAG15* was also evident in vtc1 mutants (Barth et al., 2004) while exogenous supplementation of ascorbate resoted the default expression levels consistent with wild type (Zhang, 2013b). Overall, low AsA and higher ABA promote senescence while high AsA and low ABA delays it.

Other than senescene, the role of AsA in controlling flowering, plant response to biotic stressors and in program cell death was proposed that involves a complex signal transduction network (Barth et al., 2006). Studies indicated that levels of AsA are vital in the regulation of process like senescence and flowering through complex but co-ordinated regulation of gene expression mediated by phytohormones (Barth et al., 2006). Briefly, AsA is indirectly involved in **floral induction** due to its interaction with ABA and GA production (Barth et al., 2006). It is well evident that GA promotes flowering (Yamaguchi et al., 2014; Xu et al., 2015), inhibits senescence (Currey et al., 2013), antagonizes the effect of salicylic, jasmomic acid and ABA, and shows a complex interplay among hormones in order to mediate signaling which can be in the form of a network (He et al., 2001; Navabpour et al., 2003; Barth et al., 2006).

Ascorbate is a key antioxidant molecule for sustained **photosynthesis** (Foyer, 2015). Plant mitochondrion is believed to be the major site for ascorbate biosynthesis (Wheeler et al., 1998; Smirnoff, 2000) and being a vital plant cellular component, it sometimes accumulates in chloroplasts at a concentration up to 50 mM (Foyer et al., 1983; Foyer and Noctor, 2011). Light induced accumulation of AsA has been reported in leaves of *Arabidopsis thaliana* and is attributed sustained photosynthesis through its involvement in photo-protection of light harvesting complexes (Smirnoff and Wheeler, 2000). In consistent with these reports, Yabuta et al. (2007) reported higher AsA accumulation in Arabidopsis leaves acclimated to intensive light while AsA contents decreased linearly in plants grown under low and without light. In addition to its role in leaf photo-protection, AsA is an important co-factor in photosynthetic enzymatic reactions and play a role in the synthesis of gibberellins and ethylene through specific AsA-dependant dioxygenase catalyzed reactions (Arrigoni and De Tullio, 2000; Wang et al., 2013). In chloroplasts, the role of ascorbate has been proposed during breakdown of water in thylakoid lumen while in stroma to detoxify H_2_O_2_ formed by the action of Fe-SOD and Cu/Zn-SOD (Gallie, 2012). Overall in a broader perspective, the regulation of plant development under normal and stress environment is hormone dependant, but the role of ascorbate is essential for this development to proceed.

## Role of ascorbate in chloroplast redox reactions

The biosynthesis of this vital metabolite (ascorbate) takes place in mitochondria, however, its prime requirement is in the chloroplast (Foyer, 2015). It is reported that some ascorbate-specific transport carriers ensure its diffusion to stroma from the cell cytosol (Foyer and Lelandais, 1996; Eskling and Akerlund, 1998; Horemans et al., 2000; Foyer, 2015). One such transporter has been recently reported to be present on the chloroplast envelope membrane of *Arabidopsis thaliana*, and it belongs to phosphate transporter 4 family, AtPHT4.4 (Miyaji et al., 2015). However, how ascorbate is transported from chloroplast stroma to thylakoid lumen is yet to be established (Ivanov, 2014).

The light triggered splitting of water molecule in the oxygen-evolving complex (OEC) and subsequent transfer of e^−^ from photosystem II (PSII) to photosystem I (PSI) generates ROS in chloroplasts (Trubitsin et al., 2014). Chloroplast is highly susceptible to H_2_O_2_ as it oxidizes thiol groups of chloroplast proteins (Ivanov, 2014). The H_2_O_2_ mediated suppression of key Calvin cycle enzymes is reported (Kaiser, 1979; Ivanov, 2014). In agreement, 50% suppression in the CO_2_ fixation was reported in response to 10 μm H_2_O_2_ concentration in an experiment conducted on intact chloroplast (Kaiser, 1976). Interestingly, Ivanov (2014) reported similar concentrations of H_2_O_2_ accumulated in chloroplast in an interval of every 1 s if photosynthetic electron transport chain only transfers 1% of the generated electrons (e^−^) to molecular oxygen (O_2_). This may finally result in severe oxidative stress in the light harvesting organelle and therefore the free radicals are required to be quenched.

Ascorbate peroxidases (APX) are primarily the dominant antioxidant enzymes that neutralize chloroplastic H_2_O_2_ and ensure sustained light and dark reactions (Noctor and Foyer, 1998; Trubitsin et al., 2014). Two exclusive chloroplast APX isoforms, for instance, thylakoid membrane bound form (tAPX) and stromal sAPX perform vital roles of ROS detoxification (Miyake and Asada, 1992; Foyer, 2015). Furthermore, very high ascorbate levels in the chloroplast are very closely linked with the redox status of chloroplast and activity of APX (Foyer, 1993; Smirnoff, 1996; Gest et al., 2013; Ivanov, 2014) and the reduced ascorbate pool accumulated by chloroplast is to protect itself from uncontrolled oxidation (Foyer, 2015). The APX activity is dependent on chloroplast ascorbate pool and low concentrations of the ascorbate substrate can result in irreversible inactivation of APX enzyme activity (Nakano and Asada, 1987; Foyer, 2015). Nonetheless, besides ascorbate being the APX substrate, it non-enzymaticaly neutralizes other ROS generated in the light harvesting organelle (Ivanov, 2014; Foyer, 2015).

Apart from ROS detoxification, the presence of ascorbate in chloroplast provides protection from light-induced photo-inhibition of photosynthesis (Ivanov, 2014; Miyaji et al., 2015). Zeaxanthin interaction with ascorbate is known to protect high light induced destruction of light harvesting complexes and subsequent regulation of photo-inhibition of photosynthesis through thermal dissipation (Horton and Ruban, 2005). A very important enzyme in this regard is violaxanthin de-epoxidase (VDE), commonly known as VDE (Horton and Ruban, 2005) which is located in the thylakoid lumen (Ivanov, 2014). Ascorbate is the co-factor of VDE and aids in zeaxanthin biosynthesis, as well as it participates in non-photochemical quenching and energy dissipation protecting photosystem II from photo-inhibition involving the xanthophyll cycle (Smirnoff, 2000; Müller-Moulé et al., 2002; Foyer, 2015). Due to the fact that ascorbate acts as an electron donor for the de-epoxidation of violaxanthin (Ivanov, 2014), the competition between APX and VDE for the substrate ascorbate was also evident in experiments conducted with *Spinacea oleracea* (Neubauer and Yamamoto, 1994) and *Zea mays* (Ivanov and Edwards, 2000).

Besides the above-mentioned role of ascorbate in diverse redox and ROS neutralization reactions in the chloroplast, ascorbate can be facultative electron donor for photosynthetic electron transport chain. In agreement with this, its interaction with electron transport chain (Mano et al., 2004; Ivanov et al., 2005; Ivanov, 2014) has been proposed. It is reported that under different abiotic stress conditions, suppression of water splitting complex is evident. Under these redox limiting conditions, ascorbate is proposed as an alternate e^−^ donor to PSII thereby reverting photo-inactivation (Tóth et al., 2011; Trubitsin et al., 2014). Above all, ascorbate is a multifunctional and versatile metabolite that plays essential regulatory roles in plant physio-biochemical functioning and development.

## Ascorbic acid and abiotic stress conditions

Ascorbic acid (AsA) is known to protect organelles and cells from the adversaries of ROS, which over-accumulate due to stress-induced oxidative damages (Latif et al., 2016; Mukhtar et al., 2016; Naz et al., 2016). It controls cell division and cell expansion, acts as a cofactor of many enzymes, modulates plant sense, and is involved in photosynthesis, hormone biosynthesis, and regeneration of antioxidants (Gallie, 2012; Lisko et al., 2014). Although AsA is found in different plant tissues, its levels are abundant in meristems, photosynthetic cells and different fruits (HongBo et al., 2005; Mazid et al., 2011). Higher concentration of AsA has been reported in fully developed chloroplasts of mature leaves. Under normal conditions, AsA is available mostly in reduced form (Khan et al., 2011).

In most plant species, the levels of AsA are not adequate to mitigate effectively the adverse effects of a stress (Shafiq et al., 2014; Latif et al., 2016). Thus, under such circumstances exotic application of AsA is considered advisable (Mukhtar et al., 2016; Naz et al., 2016). A summary of the results of different studies on exogenous application of AsA is presented in Table 1. These studies clearly show that external treatment of AsA is not only effective in improving yield and growth of plants by regulating a number of physio-biochemical pathways under non-stress conditions, but also under stressful cues, such as salinity, drought and temperature extremes.

High accumulation of soluble salts within plant cells/tissues can perturb the normal growth metabolism and functioning of important crop plants, particularly glycophytes when grown under saline habitats. Exogenous application of AsA through different modes has been widely reported to improve salinity tolerance of many plants (Hussein and Alva, 2014; Barus et al., 2015). For example, in an earlier study, the effects of various levels of AsA as a seed treatment (0.25, 0.5, and 1 mM) were assessed in callus culture/plants of sugarcane grown under saline conditions (Munir et al., 2013). Of all AsA pre-treatments used, generally 0.25 and 0.5 mM had a positive effect on plant growth, activities of antioxidant enzymes and soluble proteins in both plants and callus culture. However, the best response was observed at AsA pretreatment to callus cultures and *in vitro* grown plants, respectively (Munir et al., 2013). In another study, the effect of AsA was observed on the leaf growth and different physio-biochemical attributes of wheat plants under saline stress by Azzedine et al. (2011). They suggested that application of AsA was effective in alleviating the adverse effects of salinity stress by enhancing chlorophyll, carotenoids, proline accumulation, and leaf area, while decreasing H_2_O_2_ levels in plant tissues. The authors inferred that AsA can improve salt tolerance by regulating a myriad of biological processes (Azzedine et al., 2011). While observing the effect of root zone applied AsA (0.5 mM) on wilting in tomato seedlings exposed to saline stress (300 mM NaCl), Shalata and Neumann (2001) observed that application of AsA was effective in the subsequent recovery and 50% survival of the wilted seedlings. Similarly, foliar-applied AsA has also been found effective in up-regulating the activities of some key enzymes of oxidative defense system in a halophyte *Lymonium stocksii* under saline stress (Hameed et al., 2015) and enhancing macronutrient contents e. g., N, K, and P in sweet pepper subjected to salinity stress (Talaat, 2003).

Under drought stress, Azooz et al. (2013) found that seed soaking in AsA improved leaf chlorophyll, plant water status and soluble proteins in *Vicia faba* plants. Similarly, Reiahi and Farahbakhsh (2013) found that seed priming with AsA (0.5, 1.0, and 1.5 mM) improved seed germination and seedling resistance of sorghum (*Sorghum bicolor*) to drought stress. In another study with cauliflower plants, Mukhtar et al. (2016) observed that foliar applied AsA (75 and 150 mg L^−1^) was effective in improving plant growth due to AsA-induced decrease in H_2_O_2_ contents and membrane permeability and increase in chlorophyll, proline, GB, potassium and phosphorus contents under water-deficit conditions. Likewise, seed treatment with AsA improved drought stress tolerance in two cauliflower cultivars due to AsA-induced improvement in the activities of catalase (CAT) and (SOD) enzymes in cauliflower plant (Latif et al., 2016).

The role of exogenous application of AsA has been studied widely for salinity and drought tolerance capabilities but very few studies have been carried-out to assess the influence of AsA on altering the defense potential of different plants against temperature stress. In a study, foliar-applied AsA has been found very effective in improving the growth of strawberry plants under high temperature (44°C) (Ergin et al., 2014). For example, exogenously applied AsA (3 mM) increased cell turgidity of the strawberry plants and hence their growth. Foliar or seed treatment with 20 and 40 mg L^−1^ AsA improved the seedling growth and yield production of maize under low temperature stress (Ahmad et al., 2014). In an earlier study, Stevens et al. (2008) reported that the allele of the gene for monodehydroascorbate reductase (MDHAR) (which catalyzes monodehydroascorbate to ascorbate), responsible for high AsA content in fruits, is usually found active at different fruit ripening stages. Introgression of this enzyme in wild-type *Solanum pennellii* shows that MDHAR activity enhances AsA levels and improves shelf life in tomato fruit experiencing freezing stress.

Increase in intrinsic levels of AsA through genetic engineering or exogenous application can mitigate the injurious effects of environmental stresses on plants (Chen et al., 2003; Eltelib et al., 2012; Mukhtar et al., 2016). Wang et al. (2010) analyzed the physiological role of dehydroascorbate reductase (DHAR) which catalyzes the reduction of dehydroascorbate (DHA) to ascorbate in *Arabidopsis* plants under drought stress. They found that transgenic seedlings overexpressing *Myc-dhar* gene increased DHAR overexpression (1.5–5.4-fold) which increased AsA level (2–4.25-fold) and ratio of AsA/DHA (3–16-fold) relative to those in wild type *Arabidopsis* plants. They suggested that increase in plant AsA content through enhanced ascorbate recycling could limit the deleterious effects of stress-induced oxidative stress, particularly temperature stress (Wang et al., 2010).

Overall, high accumulation of AsA in plants under stress conditions as comprehensively summarized in Table 1 is linked to stress tolerance mediated by AsA scavenging ROS and upgrading the oxidative defense potential of the plants, resulting in better growth and development under stress conditions. Little information is available in the literature on how and up to what extent AsA can trigger metabolic processes operating at different phases of plant growth and development. Uncovering of this information can be helpful to fully elucidate the effective role of AsA in promoting growth under stressful cues.

## Present and future prospects

Ascorbic acid (AsA) is a vital metabolite which is essentially required for the regulation of key physio-biochemical processes in plants. As mentioned earlier, under normal conditions it contributes to a wide range of plant processes, e.g., chloroplast redox reactions, photosynthesis, seed germination, floral induction, fruit expansion, cellular ROS regulation, oxidative defense system, membrane stability, plant water status and senescence etc. Due to its multipurpose involvement within plant cells, its role in plants under stress conditions has become crucial. Several studies have reported the positive influence and stress amelioration by the exogenously supplementation of AsA on plants under various abiotic stress conditions. Varying levels of AsA have been applied during different phases of development to improve stress tolerance in different types of crops including vegetables, oil-seeds, major cereals, pulses, squash, cotton, ornamental/medicinal plants as well as sugarcane. Effectiveness of AsA in the range of 0.1–5.0 mM as well as 10–2000 mg L^−1^ as seed soaking/priming, foliage spray and through root growing medium have been assessed in different plants at different growth stages. Of all doses, it is difficult to ascertain that what dose of AsA is better, as effectiveness varied with a stage of plant growth, mode of AsA application, genetic architecture of a plant species, and type of a stress. Different growth stages of plants have been tested for their response to AsA application, but differential response of different plants has been observed. In most of the studies reported in the literature, the effect of AsA has been examined at a single growth stage, but there is a need to investigate the response of a plant to AsA at every growth stage.

In almost all studies reported so far on the role of exogenously applied AsA, synthetic/commercially available AsA have been used. However, extracts of none of the natural plants/fruits, such as papaya, bell peppers, citrus, and straw berries as sources of AsA can been tested to overcome the adverse effects of environmental adversaries on different plants through an eco-friendly source of AsA. Also as a future perspective, the intermediates mentioned in the schematic diagram could be targeted to enhance the AsA contents in plant cells/tissues and modulate subsequent plant stress responses. In this context, it should be investigated that whether the exogenous application of mannose, galactose, gulose and myo-inositol could modulate AsA pools in plant cells and subsequent response to environmental stresses. Similarly, compounds like L-gulono-1,4-lactone and L-galactono-1,4-lactone can also be targeted for improved AsA biosynthesis and investigated their role under stress conditions. Despite the considerable role of AsA in plant stress tolerance, the role of naturally occurring or synthetic analogs of AsA has rarely been focused. Very few studies are available wherein the activities of different enzymes involved in the biosynthesis of AsA and its analogs have been studied, particularly in plants exposed to different stresses. Thus, this needs to be included/elucidated in future research programs.

On a biotechnological note, over-expression of the enzymes involved in the biosynthesis of AsA can be targeted to regulate vitamin C contents of plants. There is a need to focus metabolic and biotechnological engineering for improved AsA contents among plants so as to achieve improved tolerance to abiotic stresses. Although the biosynthesis is tightly regulated, the overexpression of certain genes have been successfully achieved in several biofortification studies (Locato et al., 2013). In the past such efforts increased the AsA content in strawberry (Agius et al., 2003), potato tubers (Hemavathi et al., 2009) and cultured tobacco cells (Tokunaga et al., 2005). Similalry, significant improvement in the vitamin C content by the overexpression of some biosynthetic genes has been practically achieved in tomato, potato, lettuce and strawberry (Zhang et al., 2011; Bulley et al., 2012; Cronje et al., 2012). The transgenic plants over-expressing ascorbate biosynthetic genes exhibit enhanced oxidative stress tolerance (Zhang et al., 2011). Nonetheless, information about the strategies for bio-engineering of plant vitamin C contents has been provided in an extra ordinary detailed review by Locato et al. (2013). Furthermore, in the context of modern gene editing technologies like CRISPR-Cas9 system, plant ascorbate contents could be practically modulated, possibly better than ever before.

Another important aspect for future research is to investigate the comparative chemistry of regulation of AO and APX and how these two enzymes contribute to apoplast redox status, stress perception and subsequent downstream signaling events. Both AO and APXs are responsible for the conversion of reduced form of ascorbate to an oxidized form (dehydro-ascorbate) and which is again reduced (ascorbate) in cytoplasm by glutathione and then transported back to cell apoplast. In this context, study of apoplast redox status, hormone levels especially auxin and their relationship with NADPH oxidase and comparative regulation of AO and APX activities are essential. These could provide insight into mechanisms of plant environmental stress perception, its amplification and most importantly to discriminate between plant developmental and stress responses.

## Author contributions

NA wrote the manuscript, FS contributed in section introduction of this manuscript, while MA reviewed and updated the manuscript.

### Conflict of interest statement

The authors declare that the research was conducted in the absence of any commercial or financial relationships that could be construed as a potential conflict of interest.
